# Usefulness of Glycemic Gap to Predict ICU Mortality in Critically Ill Patients With Diabetes

**DOI:** 10.1097/MD.0000000000001525

**Published:** 2015-09-11

**Authors:** Wen-I. Liao, Jen-Chun Wang, Wei-Chou Chang, Chin-Wang Hsu, Chi-Ming Chu, Shih-Hung Tsai

**Affiliations:** From Department of Emergency Medicine, Tri-Service General Hospital, National Defense Medical Center, Taipei, Taiwan (W-IL, J-CW, S-HT); Department of Radiology, Tri-Service General Hospital, National Defense Medical Center, Taipei, Taiwan (W-CC); Department of Emergency and Critical Care Medicine, Taipei Medical University-Wan Fang Hospital, Taipei, Taiwan (C-WH); and School of Public Health, National Defense Medical Center, Taipei, Taiwan (C-MC).

## Abstract

Stress-induced hyperglycemia (SIH) has been independently associated with an increased risk of mortality in critically ill patients without diabetes. However, it is also necessary to consider preexisting hyperglycemia when investigating the relationship between SIH and mortality in patients with diabetes. We therefore assessed whether the gap between admission glucose and A1C-derived average glucose (ADAG) levels could be a predictor of mortality in critically ill patients with diabetes.

We retrospectively reviewed the Acute Physiology and Chronic Health Evaluation II (APACHE-II) scores and clinical outcomes of patients with diabetes admitted to our medical intensive care unit (ICU) between 2011 and 2014. The glycosylated hemoglobin (HbA1c) levels were converted to the ADAG by the equation, ADAG = [(28.7 × HbA1c) − 46.7]. We also used receiver operating characteristic (ROC) curves to determine the optimal cut-off value for the glycemic gap when predicting ICU mortality and used the net reclassification improvement (NRI) to measure the improvement in prediction performance gained by adding the glycemic gap to the APACHE-II score.

We enrolled 518 patients, of which 87 (17.0%) died during their ICU stay. Nonsurvivors had significantly higher APACHE-II scores and glycemic gaps than survivors (*P* < 0.001). Critically ill patients with diabetes and a glycemic gap ≥80 mg/dL had significantly higher ICU mortality and adverse outcomes than those with a glycemic gap <80 mg/dL (*P* < 0.001). Incorporation of the glycemic gap into the APACHE-II score increased the discriminative performance for predicting ICU mortality by increasing the area under the ROC curve from 0.755 to 0.794 (NRI = 13.6%, *P* = 0.0013).

The glycemic gap can be used to assess the severity and prognosis of critically ill patients with diabetes. The addition of the glycemic gap to the APACHE-II score significantly improved its ability to predict ICU mortality.

## INTRODUCTION

Emergency department (ED) hyperglycemia has been observed to be a strong predictor of in-hospital outcomes.^1^ Stress-induced hyperglycemia (SIH) is common in patients with critical illness, including sepsis, multiple trauma, major surgery, and acute myocardial infarction (AMI).^2–5^ SIH occurs secondary to an increase in the levels of counter-regulatory hormones (cortisol, catecholamines, glucagon, and growth hormone), which results in increased gluconeogenesis and decreased glycogenolysis. Notably, the phenomenon occurs in individuals with and without a history of diabetes.

The Acute Physiology and Chronic Health Evaluation II (APACHE-II) score is a commonly used model for predicting mortality in the intensive care unit (ICU). However, glucose levels are not included; despite the growing evidence of the negative effect of hyperglycemia on ICU mortality.^6^ Blood glucose levels may also reflect different severities of stress depending on whether a patient has diabetes. In patients without diabetes, not only is there evidence of a stronger association between ICU mortality and elevated levels of mean serum glucose and glucose variability^7,8^ but also the mortality risk is greater.^6^ Conversely, acute hyperglycemia in patients with diabetes could result from acute physiological stress, a high baseline blood glucose, or both, which confounds the assessments.

A strong correlation between glycosylated hemoglobin (HbA1c) and mean plasma glucose levels in the preceding 3 months was found in an international multicenter A1C-derived average glucose (ADAG) study, which allows long-term average glucose levels to be estimated using HbA1c values. We hypothesize that glycemic gap which is calculated by subtracting the ADAG from the admission glucose levels may eliminate the influence of chronic hyperglycemia on the disease severity assessments in patients with diabetes. In our previous work, we found that an elevated glycemic gap (over 72 mg/dL) was associated with adverse outcomes in diabetic patients with pyogenic liver abscess.^9^ However, it remained unclear whether the elevated glycemic gap could predict ICU mortality in patients with diabetes.

In the present study, we aim to determine whether the glycemic gap could be used to predict ICU mortality and whether incorporation of the glycemic gap into the APACHE-II score could increase the discriminative performance for predicting ICU mortality.

## METHODS

### Study Design and Setting

We conducted a retrospective observational study of consecutive patients with diabetes admitted to our 4 different medical ICUs between January 1, 2011 and December 31, 2014. Our department is an ICU of the Tri-Service General Hospital, a tertiary referral medical center in northern Taiwan. The institutional review board for human investigation approved this study and waived the need for informed consent.

### Patients

We included consecutive patients with diabetes. Diabetes was considered present if a patient was discharged from a hospital with a diagnosis of type 1 or type 2 diabetes, at least one prescription for insulin or an oral antidiabetic agent, and/or had an HbA1c level of ≥6.5% in the preceding 2 months. Patients were excluded based on the following criteria: age <18 years, hypoglycemia (blood glucose < 70 mg/dL) at initial presentation in the ED, an admission diagnosis of diabetic ketoacidosis or hyperosmolar hyperglycemic state, treatment with corticosteroids and death within 1 day of admission. Included patients were classified into several categories according to their primary diagnosis as follows: Cardiac and vascular, Thoracic and Respiratory, Neurological, Gastrointestinal, and others.^10,11^ These data were collected in an organized data collection sheet.

### Data Collection

The medical records of included patients were reviewed for the following data: age; sex; underlying comorbidities; laboratory data, including plasma glucose level at initial ED presentation and HbA1c levels (measured within 1 month before or at admission); adverse outcomes; length of mechanical ventilation; and the length of stay in the ICU and hospital. The following adverse outcomes were recorded: mortality during admission; multiple organ dysfunction syndrome; acute respiratory distress syndrome; acute respiratory failure (defined as the need for ventilatory support); failure to wean from a ventilator (defined as continued mechanical ventilation during discharge); shock (defined as persistent hypotension despite adequate fluid resuscitation); acute kidney injury (defined as serum creatinine elevated > 0.3 mg/dL or 50% from baseline); upper gastrointestinal bleeding (defined as melena with positive occult blood, bright-red blood discharge from a nasogastric tube, or endoscopic evidence of mucosal bleeding); and AMI during hospitalization.

### Measurements of Serum Glucose Levels and HbA1c Values

The admission glucose level was defined as the initial glucose recorded in the ED. HbA1c assays were performed at the Tri-Service General Hospital, using a blood analyzer (Primus CLC 385; Primus Corporation, Kansas City, MO) equipped with a high-performance liquid chromatography system.

To convert HbA1c levels to chronic average blood glucose levels, we used the following equation: ADAG = [(28.7 × HbA1c) − 46.7].^12^ The glycemic gap was calculated from the glucose level on admission, as follows: glycemic gap = [admission glucose − ADAG].

### Statistical Analysis

Continuous data are expressed as the mean ± standard deviation and categorical data are expressed as frequencies (percentage). Analyses were performed by the 2-tailed Student *t* test and the Chi-square test or Fisher exact test as applicable. A receiver operating characteristic (ROC) curve was plotted to analyze the discriminative power of the prediction tools, and the area under the ROC curve (AUC) and 95% confidence internal (CI) was calculated. The log-rank test was used to determine the statistical significance on survival curves. The net reclassification improvement (NRI), a function of MATLAB (MathWorks, Natick, MA), was used to assess the improvement in model performance after adding parameters.^13^ Otherwise, data were analyzed using SPSS statistics for Windows, Version 17.0 (SPSS Institute, Inc., Chicago, IL). Differences with *P* values of <0.05 were considered statistically significant.

## RESULTS

### Study Population and Baseline Characteristics

We initially identified 582 diabetic patients who were admitted to the ICUs during study period. Sixty-four patients were excluded patients due to hypoglycemia (n = 17), diabetic ketoacidosis/hyperosmolar hyperglycemic syndrome (n = 26), treatment with steroid (n = 15), death within 1 day of admission (n = 3), and admission stay longer than 180 days (n = 3). We enrolled 518 patients admitted to 4 different medical ICUs, of which 87 (17.0%) died during their ICU stay. Most diagnoses were from the Thoracic and Respiratory category (38.8%), and 59.5% of all patients had accompanying infections. Compared with survivors, nonsurvivors tended to be older and to have higher rates of infections, malignancy, and mechanical ventilation (Table 1); nonsurvivors also had higher APACHE-II scores, admission glucose levels, maximum glucose levels (first 48 h), mean glucose levels (first 24 h), and glycemic gaps (*P* < 0.001, Table 2). However, there was no significant difference in HbA1c values between survivors and nonsurvivors.

**TABLE 1:**
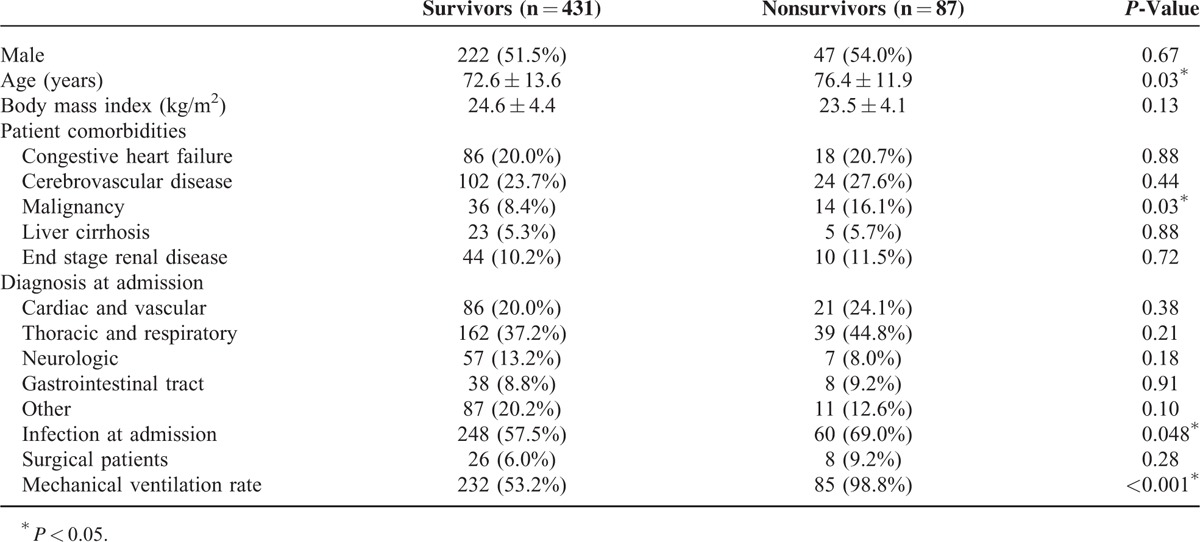
Underlying Conditions of the Diabetic ICU Survivors and Nonsurvivors

**TABLE 2:**
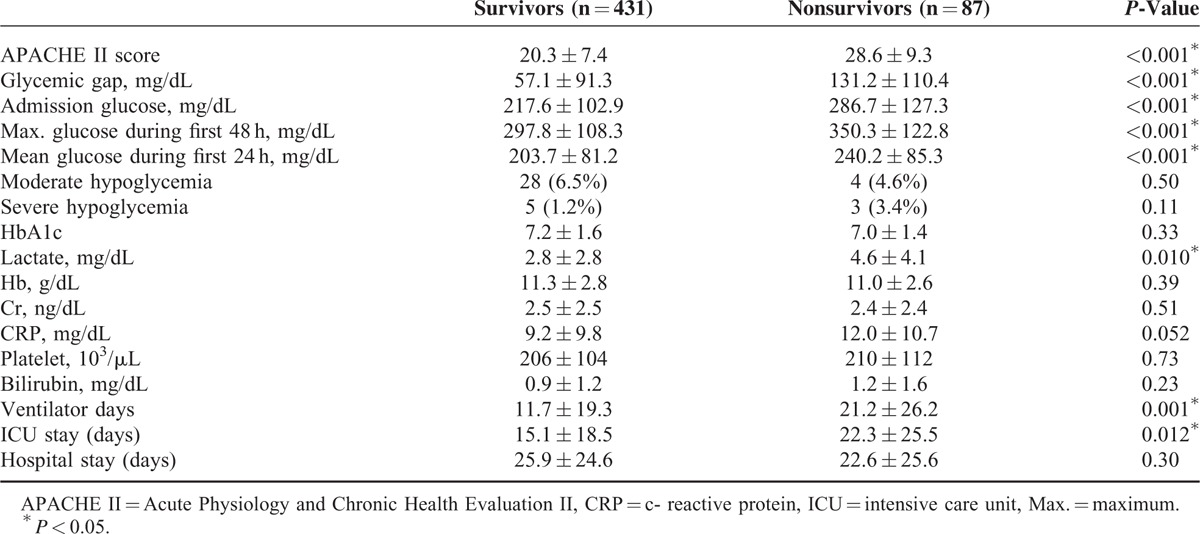
Comparison of the Characteristics of the Diabetic ICU Survivors and Nonsurvivors

### Predictors of ICU Mortality

**FIGURE 1:**
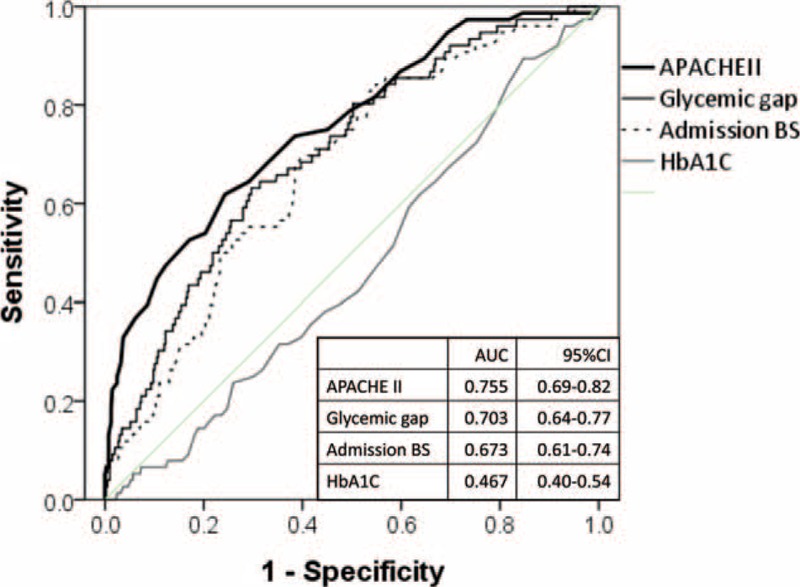
ROC curves for glucose parameters and the APACHE-II score for predicting ICU mortality. Glycemic parameters included admission glucose levels, glycemic gap, and HbA1c. The AUC of the APACHE-II score was larger than that of glycemic gap or admission glucose levels (*P* < 0.001).

Figure 1 summarizes the AUCs for key predictors of ICU mortality. The APACHE-II score had the highest AUC (=0.756, 95% CI: 0.69–0.82). The discriminative power of the glycemic gap for ICU mortality was greater than that of admission glucose, maximum glucose (first 48 h), and mean glucose (first 24 h) levels, with AUCs of 0.703 (95% CI: 0.64–0.77), 0.673 (95% CI: 0.61–0.74), 0.625 (95% CI: 0.55–0.70), and 0.635 (95% CI: 0.57–0.70), respectively (*P* < 0.001).

### Glycemic Gap in Critically Ill Patients With Diabetes

**FIGURE 2:**
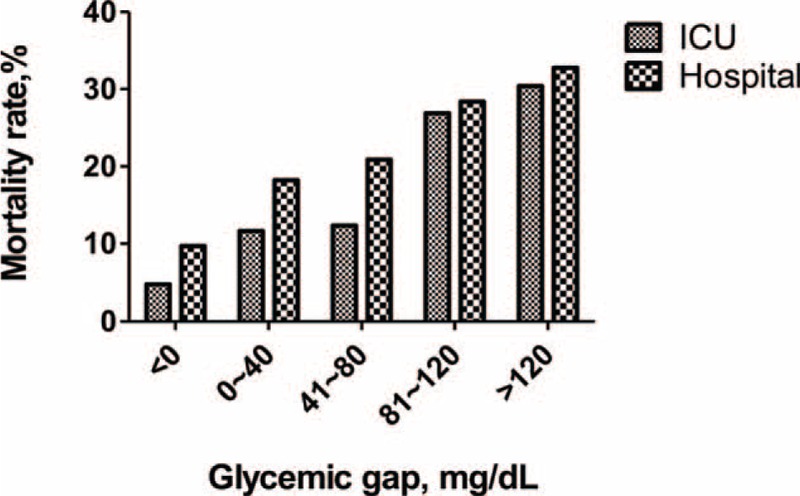
ICU and hospital mortality according to glycemic gap categories among critically ill patients with diabetes. There was an upward trend for both ICU and hospital mortality with increasing glycemic gaps. The ICU mortality rate increased markedly when the glycemic gap exceeded 80 mg/dL.

**FIGURE 3:**
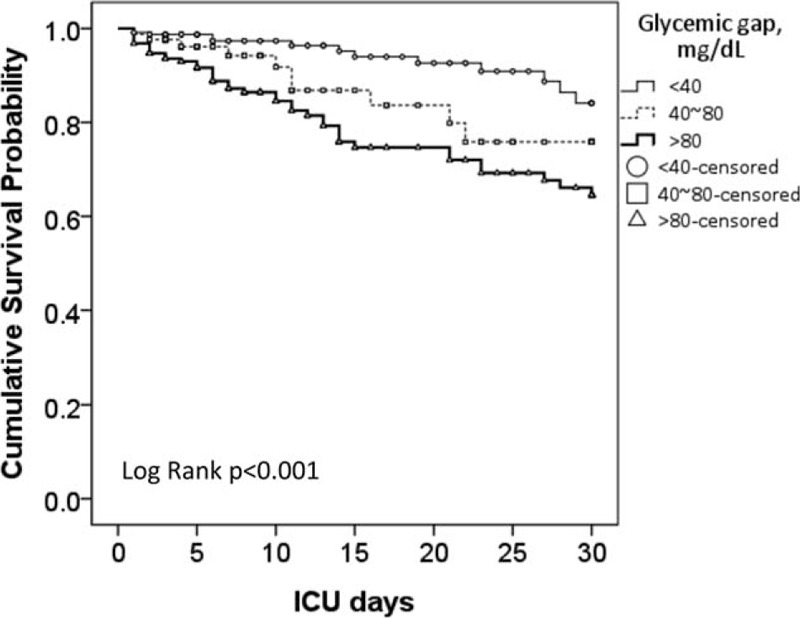
Kaplan–Meier survival curve of the glycemic gap in critically ill patients with diabetes. The ICU mortality was statically significant between diabetic patients with high (>80 mg/dL), intermediate (40–80 mg/dL), and low (<40 mg/dL) glycemic gaps.

**FIGURE 4:**
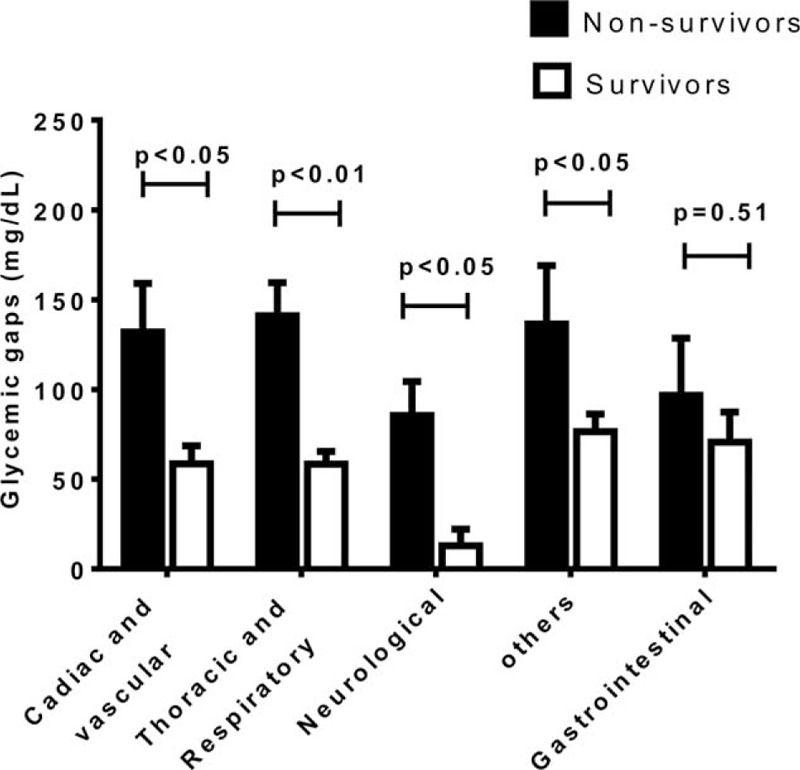
The association between glycemic gaps and mortality in different ICU admission categories ICU survivors who were categorized to Cardiac and vascular, Thoracic and Respiratory, Neurological, and others subgroups had statistically significant lower glycemic gaps than nonsurvivors.

The optimal cut-off for the glycemic gap to predict ICU mortality in patients with diabetes was ≥80 mg/dL (using the Youden index), which provided a sensitivity and specificity of 64.0% and 68.8%, respectively. Patients with a glycemic gap ≥80 mg/dL had significantly higher ICU and hospital mortalities and higher incidences of major complications compared with patients who had a glycemic gap <80 mg/dL (Table 3). Patients with higher glycemic gaps tended to have increased risks of ICU and hospital mortalities (Figure 2). The ICU mortality rate increased markedly when the glycemic gap exceeded 80 mg/dL. The Kaplan–Meier survival curve shows that a glycemic gap >80 mg/dL was associated with a significantly shorter survival than glycemic gaps of 40–80 mg/dL and <40 mg/dL (Figure 3). As shown in Figure 4, compared to nonsurvivors, ICU survivors who were categorized to Cardiac and vascular (*P* < 0.05), Thoracic and Respiratory (*P* < 0.01), Neurological (*P* < 0.05), and others (*P* < 0.05) subgroups had statistically significant lower glycemic gaps than nonsurvivors, but not those categorized to gastrointestinal.

**TABLE 3:**
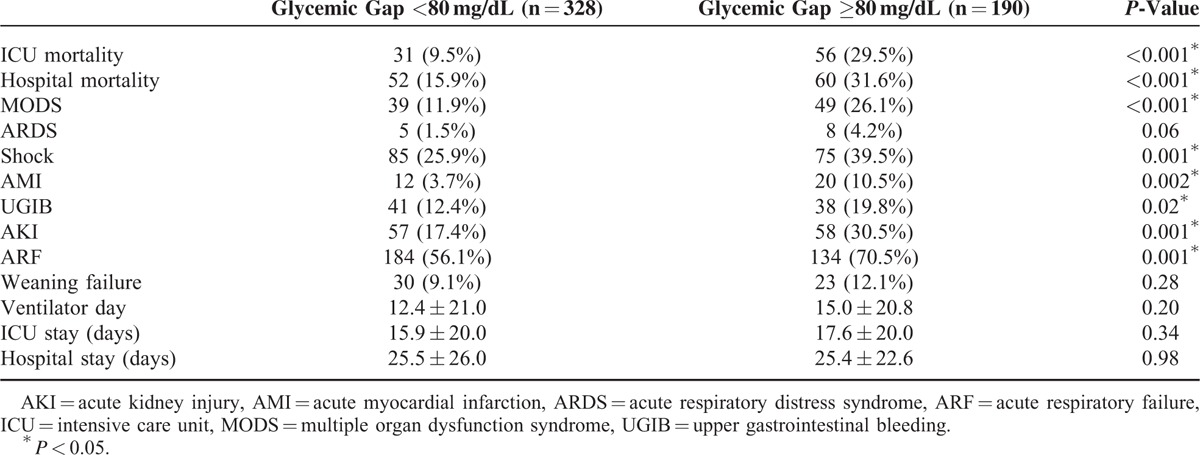
Clinical Outcome Versus Glycemic Gap of Diabetic ICU Patients

### Incorporating the Glycemic Gap Into the APACHE-II Score

**FIGURE 5:**
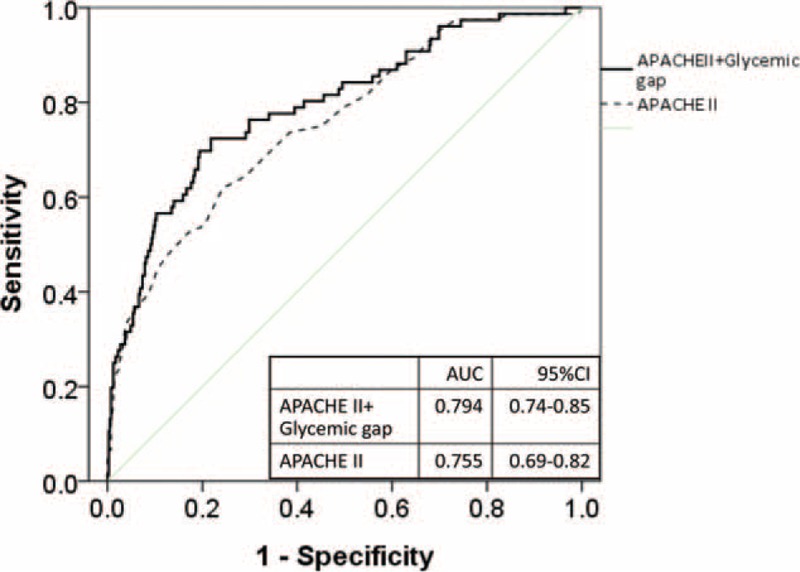
ROC curves after integrating the glycemic gap into the APACHE-II score. Combining the glycemic gap with the APACHE-II score significantly increased its discriminative ability to predict ICU mortality, increasing the AUC from 0.755 to 0.794 (NRI = 13.6%, *P* = 0.0013).

Incorporating the glycemic gap into the APACHE-II score increased its discriminative performance for predicting ICU mortality by increasing the AUC from 0.755 (95% CI: 0.69–0.82) to 0.794 (95% CI: 0.74–0.85) (NRI = 13.6%, *P* = 0.0013, Figure 5).

## DISCUSSION

Our major findings in patients with diabetes are as follows: compared with other blood glucose-based parameters, the glycemic gap was able to predict ICU mortality; a glycemic gap ≥80 mg/dL was associated with significantly higher ICU and in-hospital mortality rates and adverse outcomes compared with those with a glycemic gap <80 mg/dL; and adding the glycemic gap to the APACHE-II score could significantly increase its discriminative power. Thus, the glycemic gap could be successfully incorporated into future clinical scoring systems to enhance their discriminative performance.

Researchers have suggested that SIH could predict outcomes in critically ill patients because the severity of SIH correlates to disease severity. SIH forms a part of the adaptive response to critical illness, in which excessive cytokine and counter-regulatory hormone release results in insulin resistance. Hyperglycemia and insulin resistance could be evolutionarily preserved responses that increase the chances of survival from acute illness. Therefore, attempts to interfere with this exceedingly complex multisystem adaptive response could be harmful.^14^ On the other hand, because hyperglycemia is the cardinal feature of diabetes, preexisting hyperglycemia must be considered when investigating the association between SIH and adverse outcomes in patients with diabetes. When acutely ill, the epiphenomenon of admission hyperglycemia could result from a combination of acute physiological stress or higher baseline blood glucose (HbA1c and ADAG).^15^ Because of the discord between these phenomena, the fundamental question with regard to acute hyperglycemia in nondiabetic and diabetic patients is complicated. We therefore speculated: what was the major determinant of elevated serum glucose in critically ill patients with diabetes? Consistent with our previous study, we observed that an elevated glycemic gap (≥80 mg/dL) could predict several adverse outcomes and ICU mortality in this patient group.^9^

We also confirmed that the APACHE-II score was a good predictor of ICU mortality in critically ill patients with diabetes than either the glycemic gap or the admission glucose level. It is unlikely that any single biochemical variable would have a sufficiently high AUC to be useful for early prognostication when used in isolation. When a novel biomarker becomes available to facilitate risk prediction, it should be compared against existing best practice tool.^16^ By incorporating the glycemic gap into the APACHE-II score, we yielded better discriminative performance for predicting ICU mortality in our cohort. The American Diabetes Association recommends biannual evaluation of HbA1c levels in patients with stable treatment and glycemic control and recommends quarterly evaluation in patients with changes in therapy or who are not meeting glycemic targets.^17^ We believe that incorporating the glycemic gap into other acute assessment tools is clinically feasible and could provide increased discriminative performance in critically ill patients with diabetes, without the need for additional laboratory examinations. However, a larger prospective cohort study is needed to confirm our hypothesis.

The difference between the ICU and hospital mortality rates was smaller for patients with a glycemic gap ≥80 mg/dL than for those with a glycemic gap <80 mg. We speculate that patients with the former had greater disease severity than the latter. A high glycemic gap in the ICU was therefore associated with less chance of surviving to the general wards.

Our results are consistent with previous studies where admission glucose, mean glucose, and maximum glucose levels were associated with adverse outcomes and ICU morality.^10,18,19^ In addition, higher admission APACHE-II scores among critically ill patients with SIH.^2^ For example, admission glucose levels, mean glucose levels, and glucose variability (first 24 h) were significantly associated with ICU mortality in critically ill patients, with similar AUCs for each glycemic variable (0.55, 0.58, and 0.58, respectively).^10^ A recent study of 194,772 patients showed that ICU mortality increased progressively with the severity of hyperglycemia,^18^ whereas another large multicenter study showed that admission hyperglycemia was associated with increased ICU mortality, including in patients with AMI, arrhythmia, unstable angina, and pulmonary embolism.^6,19^

The presence of preexisting hyperglycemia in critically ill patients may be a confounding factor for predicting ICU mortality in patients with diabetes; indeed, several studies have reported a relatively weak relationship.^6–8,20^ Egi et al observed that ICU mortality was not strictly associated with the diabetes, but with the chronic blood glucose control. They observed a stronger association between acute hyperglycemia and ICU mortality in patients without diabetes. However, poorer glycemic control has also been shown not to be associated with poorer outcomes.^10,11,21^

In 1 study, an independent association did not exist between hyperglycemia and mortality once lactate levels were considered.^22^ In a prospective multicenter study, admission hyperglycemia predicted 30-day mortality in 816 nondiabetic patients with ST-segment elevation AMI and cardiogenic shock but not among patients with diabetes.^23^ ICU mortality has been predicted by conventional disease severity factors, including APACHE-II scores, requirement for mechanical ventilation and lactic acidemia but not by maximum glucose levels.^24^ Patients with SIH due to septic shock have also been shown to have a significantly lower mortality rate than those with normal blood glucose levels.^25^ In addition, among patients with chronic obstructive lung disease treated with corticosteroids who developed significant hyperglycemia, the increase in blood glucose levels did not correlate with the maximum corticosteroid dose, mortality, length of hospital stay, or readmission rates.^26^ Moreover, neither the duration of hyperglycemia nor the amount of insulin administered affects the outcomes of patients with primary neuromuscular ARF.^27^

An explanation for the discordant results in the existing literature could be the failure to consider chronic blood glucose levels in patients with diabetes. Using the glycemic gap, we were able to eliminate the possible influence of chronic hyperglycemia in patients with diabetes.

## LIMITATIONS

Our study has several limitations. First, it was retrospective and may have been subject to selection bias. Notably, different management approaches between physicians may have influenced the study outcomes. Second, the adequacy of glycemic control during hospitalization may be relevant. During this study, the trigger to start insulin therapy was a blood glucose level of 180 mg/dL, and we did not specifically address the effects of glycemic control during hospitalization. Nonetheless, recent studies suggest that attempts at tight glycemic control do not improve outcomes.^14^ Future studies need to control for this factor in a subgroup analysis in light of the findings. Third, we did not specifically address the level of parenteral nutrition or the catecholamine dose.

## CONCLUSIONS

In this study, an elevated glycemic gap was associated with an increased risk of ICU mortality and it improved the discriminative performance of the APACHE-II score. The glycemic gap can be used to assess the severity and prognosis of patients with diabetes presenting with critical illness.

### Key Messages

1. Compared with other blood glucose-based parameters, the glycemic gap could predict ICU mortality in patients with diabetes.
2. A glycemic gap ≥80 mg/dL in patients with diabetes was associated with significantly higher ICU and in-hospital mortality rates and adverse outcomes compared with those with a glycemic gap <80 mg/dL.
3. The discriminative power of the APACHE-II score could significantly increased after adding the glycemic gap to it.
4. Glycemic gaps can be successfully incorporated into future clinical scoring systems to enhance their discriminative performance, but further prospective research is needed.
